# Communications

**Published:** 1867-07

**Authors:** 


### *Communications*

Will speedily appear from Drs. Prince, Anderson, Durham, Ormsby, Robbins, Hunt, Hart, and many others. This month permits only acknowledgement.

